# Simulation of Wood Polymer Composites with Finite Element Analysis

**DOI:** 10.3390/polym15091977

**Published:** 2023-04-22

**Authors:** Satya Guha Nukala, Ing Kong, Akesh Babu Kakarla, Vipulkumar Ishvarbhai Patel, Hossam Abuel-Naga

**Affiliations:** 1 Department of Engineering, School of Computing, Engineering and Mathematical Sciences, La Trobe University, Bendigo, VIC 3552, Australia; s.nukala@latrobe.edu.au (S.G.N.); a.kakarla@latrobe.edu.au (A.B.K.); v.patel@latrobe.edu.au (V.I.P.); 2 Department of Engineering, School of Computing, Engineering and Mathematical Sciences, La Trobe University, Melbourne, VIC 3086, Australia; h.aboel-naga@latrobe.edu.au

**Keywords:** WPCs, ANSYS, finite element analysis, stress, strain, simulation

## Abstract

Wood is a cellulosic material that is most abundantly available in nature. Wood has been extensively used as reinforcement in polymer composite materials. Wood polymer composite (WPC) is an environmentally friendly and sustainable material exploited in building and construction within the marine, packaging, housewares, aerospace, and automotive industries. However, the precision of testing equipment for finding the properties of WPCs becomes less feasible compared to experimental analysis due to a high degree of differences in the measurement of properties such as stress, strain and deformation. Thus, evaluating the mechanical properties of WPCs using finite element analysis (FEA) can aid in overcoming the inadequacies in measuring physical properties prior to experimental analyses. Furthermore, the prediction of mechanical properties using simulation tools has evolved to analyze novel material performance under various conditions. The current study aimed to examine the mechanical properties of saw dust-reinforced recycled polypropylene (rPP) through experimentation and FEA. A model was developed using SolidWorks, and simulation was performed in ANSYS to predict the mechanical properties of the WPCs. To validate the obtained results, the simulated static tension test results were confirmed with experimental tension tests, and both assessments were well in accordance with each other. Using FEA to predict material properties could be a cost-effective technique in studying new materials under varied load conditions.

## 1. Introduction

Wood polymer composites (WPCs), introduced in the 1990s, have a lesser environmental impact and lower maintenance compared to other non-sustainable glass- and carbon-reinforced composite materials [1]. Following a rapid increase in demand for sustainable materials in recent years, WPCs have attracted increasing attention from the scientific community and industries to replace non-sustainable composites [2]. The benefit of WPCs is that they possess unique characteristics such as stiffness, lower water intake, high sustainability, dimensional stability and specific strength over their lifetime, and durability against environmental impacts [3,4,5] due to their combination of wood and polymeric materials [6]. In addition, polymer composites produced using wood filler have favourable mechanical properties and a higher rigidity than unfilled polymer materials [7]. The most significant application of WPCs is in the building and construction industry [1,8]. WPCs are used in a wide range of products, such as railing and fencing for construction [9], and in the interior and exterior parts of automobiles [10,11]. Furthermore, there are some major WPC manufacturers in Australia, such as Tuff Deck—Composite Decking (Dandenong South, VIC, Australia), ModWood Technologies (Campbellfield, VIC, Australia) and Advanced Plastic Recycling (Edinburgh, SA, Australia). Prior to this, many researchers have used virgin polymers for the development of WPCs, such as polyethylene terephthalate (PET) [12], polypropylene (PP) [13,14], polyethylene (PE) [13,15], polylactic acid (PLA) [16], polyvinyl chloride (PVC) [17] and polyurethane (PU) [18], which are the most predominantly used materials as the polymer matrices in WPCs. Among the above-mentioned polymers, polypropylene (PP) reinforced with wood fillers has been used for various industrial applications extensively [19,20,21]. Over the years, the amount of plastic waste generated has been increasing and has resulted in significant amounts of municipal solid waste (MSW). Therefore, there have been attempts to recycle post-consumer plastics for the production of WPCs in order to offload their ecological impacts [22,23].

The production and usage of new composite materials should satisfy the requirements in individual industrial applications. Prototyping requires extensive trials to ensure that appropriate performance is obtained throughout its lifespan. Therefore, rather than performing a series of tests for each prototype, it is preferable to use a model that can mimic similar behaviours and conditions during simulation. This facilitates the quick correction of the digital model, evaluates results and predicts trends. Finite element analysis (FEA) is the most used simulation process among the research by the science and engineering communities [24]. Using FEA simulation to predict mechanical properties during the design phase is an effective tool for optimizing parameters.

In this context, Dickson et al. [25] reported the mechanical performance of carbon, Kevlar and glass fiber-reinforced nylon composites using FEA. The results showed that the strength of Kevlar-reinforced nylon had the highest strength followed by glass fiber- and carbon fiber-reinforced composites. Caminero et al. [26] validated the impact and damage resistance of 3D printed thermoplastic reinforced with Kelvar fibers using the fused deposition modelling technique. It was reported that the impact strength increased as the percentage of fibers increased. Alharbi et al. [27] studied the uniaxial stress–strain response of 3D printed polylactic acid (PLA) using FEA and validated it with experimental data. The results showed that the stress and displacement did not vary significantly within the element range of interest. The model showed a maximum resultant displacement of 2.227 mm and strain rate of 1.615 × 10^−2^ [27], respectively. Bhandari et al. [28] created a 3D printed model composed of polyetherimide material to predict the elastic response of the materials using FEA. The accuracy of the FEA using Poisson’s ratio was less accurate and showed a difference of up to 19.6% between the predicted and observed values. The internal lattice structure from the analysis significantly matched the 3D printed model values.

In addition, a small number of investigations has been conducted on WPCs using FEA. For instance, Roy et al. [1] demonstrated the mechanical analysis of composites made of wood waste and recycled polypropylene. The predicted flexural and tensile properties of the biocomposites were validated by experimental data. The results showed good agreement between the simulation and empirical data. Similarly, Bhaskar et al. [29] reported the results of a three-point bending test of WPCs produced from recycled polymer and pine wood flour. The recycled polymer matrix consisted of 80% polypropylene and 20% high-density polyethylene. The results showed that the composite at 40% of pine wood flour exhibited a maximum shear stress of 10.085 MPa and maximum equivalent stress of 44.815 MPa. Moreover, maximum deflection was observed at the point of contact of load. In another study, Ezzaraa et al. [30] demonstrated the FEA of 3D printed wood–PLA composites and compared the results with experimental data. The analysis was carried out to predict the mechanical properties of the 3D printed WPCs and to propose an efficient numerical tool to understand the effects of the internal porosity of 3D printed composites. The study reported that the elastic properties of the composites mostly varied based on the wood volume fraction and Young’s modulus was based on the addition of wood particles. The comparison between the FEA and experimental results showed that the predictions were significantly equal. Moreover, the analysis also concluded that wood particle size and shape were considerably subjective to the anisotropy of the composite which could enhance the longitudinal Young’s modulus and reduce the transverse Young’s modulus. Hartmann et al. [31] reported a numerical analysis of WPC using RVE and FEM in ANSYS Workbench V 21.1 (Canonsburg, PA, United States). The study was conducted using an FEA model developed based on a softwood tracheid mainly consisting of pine wood with 12% moisture at a temperature of 23 °C. The findings of the study stated that 10 MPa must be applied for the deformation of the softwood composites. However, experimental studies reported that pine wood compressive strength ranges from 4 to 14 MPa. Hence, analysis with the RVE approach was determined successfully with appropriate accuracy.

The present study aimed to create a microscale simulation model and perform the mechanical testing of WPCs using ANSYS Workbench. The software was used to simulate the layer-based composites. Furthermore, the mechanical properties of the WPCs composed of recycled polypropylene reinforced with sawdust were studied experimentally. The predicted results from the FEA were validated by the experimental data.

## 2. Materials and Methods

### 2.1. Materials

Recycled polypropylene (rPP) was collected from the Bendigo Recycling Centre, Eaglehawk, VIC, Australia. The collected rPP originated from used milk and yoghurt bottles. Sawdust (SD) was obtained from Raw Boards Pty. Ltd., Bendigo, VIC, Australia. The SD comprised different types of hardwood species (red gum, ironbark and yellow gum wood) collected from the various construction and demolition (C&D) activities with different particle sizes. The chemicals sodium stearate (C_18_H_35_NaO_2_) and sodium hydroxide (NaOH) were purchased from Bunnings, Bendigo, VIC, Australia. Hydrochloric acid (HCl) was procured from Sigma-Aldrich Pty. Ltd., Melbourne, VIC, Australia.

### 2.2. Methodology

Initially, rPP was shredded using a plastic crusher (DongGuan ZhongLi Instrument Technology Co., Ltd., Dongguan, China), and it was further cleaned with NaOH solution (5%) for 60 min. Afterwards, the rPP was washed using sodium stearate twice, followed by rinsing with water for the removal of excess dirt and other debris. The SD waste was collected from the various C&D activities in various sizes. Furthermore, the SD was sieved according to the ASTM E11 sieve method to obtain 0.05 mm particles [32]. Later, the SD was washed with a 20% NaOH solution and followed by 10 M HCl to remove excess dirt, alkaline and residues on the surface of the SD. Furthermore, the SD was rinsed three times with deionized water and oven-dried for 24 h at 70 °C as per the methodology reported by Medupin et al. [33]. Once the cleaning and segregation processes of rPP and SD were completed, they were pre-mixed in a Ziploc bag according to the concentrations shown in Table 1. The pre-mixture was later fed into a co-rotating batch mixer (ZL-3011 Rubber Lab Banbury Kneader Mixer; DongGuan ZhongLi Instrument Technology Co., Ltd., Dongguan, China). The mixer parameters, such as the hopper temperature (190 °C) and spindle speed (8 rpm), were maintained as constant and the hopper was rotated clockwise and anti-clockwise to obtain a good consistency of the WPCs. Finally, the obtained composite was crushed into tiny pieces with a plastic crusher (ZL-9031 Digital Crushing Strength Tester; DongGuan ZhongLi Instrument Technology Co., Ltd., Dongguan, China) and hot-pressed (CY-PCH-600D Laboratory Hydraulic Press; Zhengzhou CY Scientific Instrument Co., Ltd., Zhengzhou, China) at 190 °C with a pressure of 20 kPa for 15 min and cooled to room temperature [34,35]. Dog bone specimens were produced with an ASTM D638 type IV standard [36,37]. The experimental work used in determining each specimen’s mechanical properties was performed by Nukala et al. [34]. The averages of the data from the experimental work were evaluated for three replicates of each dataset using statistical analysis. The analysis was carried out with GraphPad Prism 9.0 (GraphPad Software, Inc., San Diego, CA, USA) through the ANOVA method.

### 2.3. Tensile Test

Hot-pressed dog bone specimens with a thickness of 3 mm were used for tensile testing. A Zhongli ZL-8001A tensile tester (DongGuan ZhongLi Instrument Technology Co., Ltd., Dongguan, China) at a crosshead speed of 3 mm/min with a load of 500 kN was used [38,39]. The samples were stored at a room temperature under dry conditions before testing [40,41,42].

### 2.4. Microstructural Analysis

The microstructural analysis of the fractured cross-sectional surfaces of the composites was conducted using a benchtop SEM (Hitachi Benchtop SEM 3030; Tokyo, Japan) [1]. The samples were sputter coated with platinum at 10 kV for 30 s before the examination [40]. The micrographs were obtained under variable pressures ranging from 10 to 15 kV.

### 2.5. Simulation

**Figure 1:**
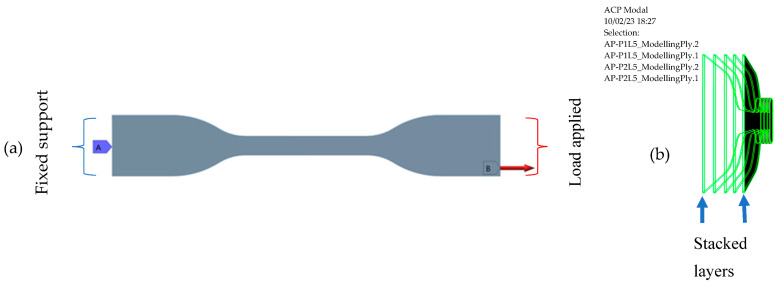
(**a**) Boundary conditions of the specimen and (**b**) stacking sequence of the specimen.

The 3D model was developed using SolidWorks software V 21.0 (Dassault Systèmes SE, Vélizy-Villacoublay, France) as per the ASTM D638 standard for tensile tests. The specimen boundary conditions were applied with one end of the model fixed (A) and the other with a load applied (B), as shown in Figure 1a. A load of 500 kN was used to predict the maximum stress, strain and deformation. The model was imported into the ANSYS Composite PrepPost (ACP) of ANSYS Workbench (Canonsburg, PA, USA) [31,43]. The ACP plugin was used to analyze the layered composites, as shown in Figure 1b. The model was stacked up in five layers with a total thickness of 3 mm. The materials used in the simulation were SD and rPP. Additionally, a customized material library was created in ANSYS Workbench using experimental data obtained from the literature [44,45,46,47]. The properties of the rPP and SD are listed in Table 2.

Based on a study by Roy et al. [1], it was assumed that the material would behave as isotropic in nature; it was further required to define its elastic coefficients in ANSYS. Therefore, the acquired engineering stress and strain values were converted into true stress and strain values according to Equations (1) and (2) [51] and the plastic strain values were calculated according to Equation (3) [1].

(1) $$\sigma_{true}=\sigma_{engineering}\times \left(1+\in_{engineering}\;\right)$$

(2) $$\in_{true}\;=\;\ln\left(1+\in_{engineering}\right)$$

(3) $$\in_{plastic}\;=\;\in_{true}-\frac{\sigma_{true}}{E}\;$$

where ∈ is the strain, σ is the stress and *E* is the Young’s modulus of the material. The engineering stress (σ*_engineering_*) and engineering strain (∈*_engineering_*) values are the experimental tensile test values [52]. The average Young’s modulus of the composite material was calculated according to Hooke’s law, i.e., stress is directly proportional to strain.

The calculated values from Equations (1) and (3) were used to define the isotropic plastic hardening characteristics in the simulation software. The parameters for the deformation rate used in the FEA were the same as per the experimental conditions. To achieve accurate results, mesh generation and element size were adjusted accordingly. The simulation results were in accordance with the experimental results since all the constraints and boundary conditions were similar. Therefore, it could be concluded that material definition was accurate.

## 3. Results and Discussion

### 3.1. Morphology

**Figure 2:**
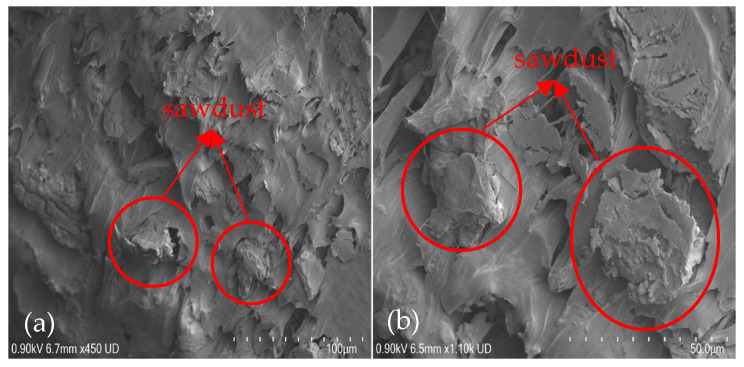
SEM micrographs of WPC samples at cross-sectional failure depicting the random distribution of SD for (**a**) rPP-SD3 and (**b**) rPP-SD4.

The micrographs of the rPP-SD composites are shown in Figure 2. It can be seen that the SD was randomly distributed in the rPP, with no clear gap, no significant alignment in a particular direction and good interfacial bonding. The following bonding is an indication of the isotropic behaviour of the composite and stress transfer from the weaker matrix to the stronger SD fiber as reported by Adhikary et al. [53]. Furthermore, Renner et al. [54] reported that the strength and interfacial interactions confirmed the composite failure mode and micromechanical deformation.

### 3.2. Mesh Generation

**Figure 3:**
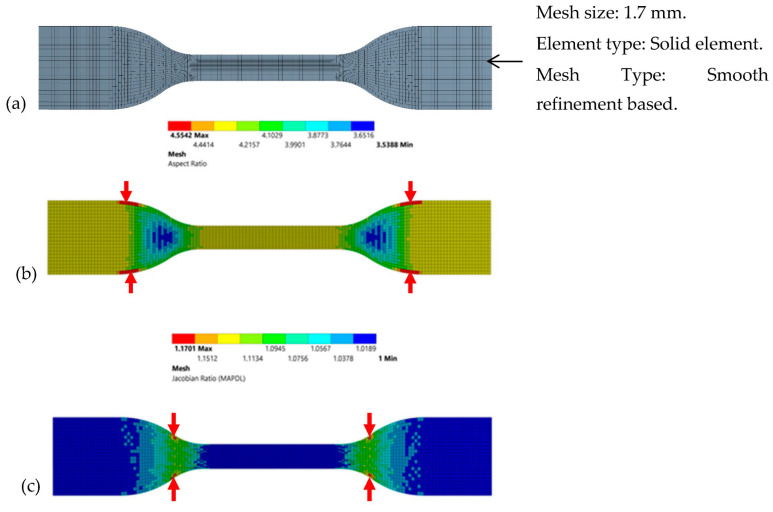
(**a**) Mesh generated in ANSYS, (**b**) mesh aspect ratio of WPCs and (**c**) Jacobian ratio of WPCs.

Mesh sensitivity is a reference for how much a solution can be changed in terms of the entities such as mesh density, element type, number of elements and nodes used for the individual problem under study [55]. The most commonly used sensitivity is type I sensitivity in FEA, and to obtain an accurate solution, a finer mesh is preferred. Alternatively, type II sensitivity is another type of mesh that can be used as a refined mesh [56,57]. As per the discussion, an evaluation of mesh sensitivity was also performed in the present study to assess the effect of mesh size on von Mises stress [58] and the resultant displacement. Smooth refinement-based mesh was chosen over the standard mesh, as shown in Figure 3a. As a result, more nodes allowed the composite specimens to obtain highly accurate results and perform large deformations [59]. Furthermore, to improve the analysis of the mesh quality, mesh plots were created. It can also be noted that the maximum stress point was located on the rim of the specimen and failed nodal points are shown with red arrows in Figure 3b,c. It is worth mentioning that there were no failed nodal points at the centerline of the specimen where necking occurred which shows that the type I meshing obtained was very fine and smooth [60]. The subsequent results proved that the mesh quality was acceptable, as the aspect ratio (Figure 3b) was less than five and the Jacobian ratio (Figure 3c) was less than two [61,62]. The Jacobian ratio measures the shape of the given element, which is compared to the ideal element, and the ideal shape of an element depends on the element type [63]. Furthermore, the ideal Jacobian ratio should be between 1 and 10 for most elements, which correlates with the present Jacobian result [64,65]. Moreover, the results indicate that the element edge lengths were close to each other and had more positive values. In addition, the mesh generated a maximum number of nodes, as displayed in Figure 3b,c [27,66].

### 3.3. Mechanical Properties

#### 3.3.1. Stress and Strain Distributions

**Figure 4:**
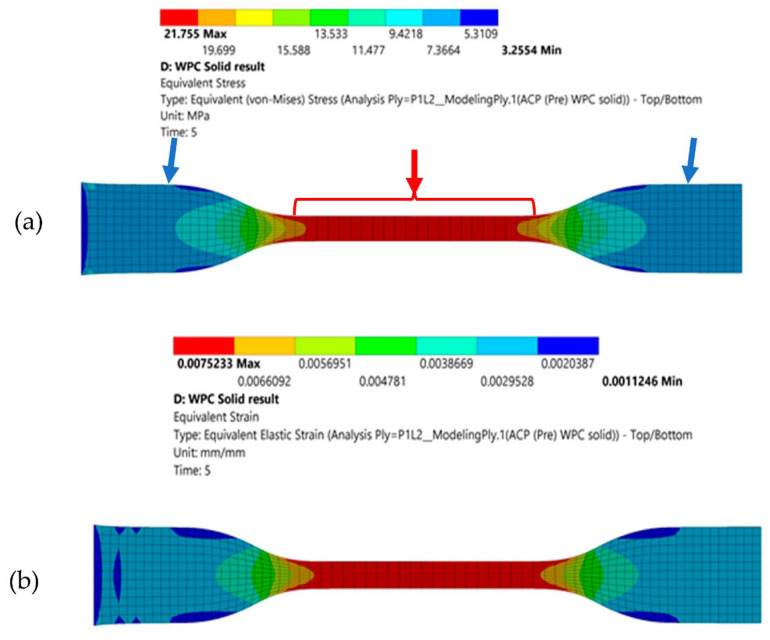
(**a**) Equivalent stress distribution and (**b**) equivalent strain distribution.

The specimen’s equivalent stress and strain distribution while being stretched is displayed in Figure 4a,b. Concentration areas of maximum and minimum stress are indicated with red and blue arrows in Figure 4a. The analysis showed that the specimens failed at a maximum stress region of 21.75 MPa and a maximum strain region of 0.0075 mm at a strain rate of 29.5%.

**Figure 5:**
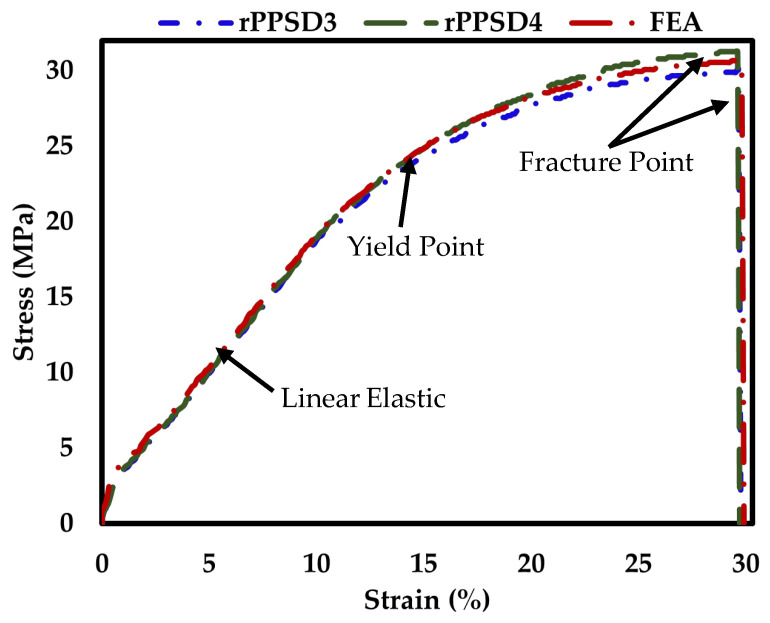
Stress–strain curve from the FEA and experimental data.

The stress–strain curve from the experimental data and FEA are shown in Figure 5. The maximum experimental stress in rPP-SD3 and rPP-SD4 was obtained at 21.65 MPa and 23.15 MPa, respectively. The maximum strain rate was approximately 29.5% in both cases. The stress–strain curve for the experiment and FEA showed a linear elastic region at the beginning of tension, followed by a yield point indicating buckling and collapse. Furthermore, the composites showed rupture and decreased stress after the yield point. The high concentration region corresponded to composite failure at a maximum stress region 21.75 MPa for FEA and 22.85 MPa for the experimental analysis. According to the studies conducted by Bhaskar et al. [29] and Tiwari et al. [67], tensile strength and durability highly depend on a specimen’s parameters such as its dimensions, thickness and pressure loading. Another study conducted by Das Lala et al. [68] demonstrated the utilization of biodegradable polymer composites such as rubber seed shell and epoxy resin. The obtained results of the composites were validated using both experimental results and FEA. The results stated that with an increase in the filler content, the tensile strength of the composite increased. Furthermore, it was stated that a similar trend was observed in the results from the finite element technique. Navaneethakrishnan et al. [69] studied the structural analysis of a natural fiber-reinforced polymer matrix composite using vinyl ester resin, sisal and luffa fiber. The results indicated that a 20% sisal and 10% luffa natural fiber composite showed higher tensile strength. The experimental results were validated against ANSYS results and the tensile strength of the natural fiber-reinforced composite was 0.05 MPa higher than the experimental result. Thus, in relation to the above-mentioned discussion, it was evident that rPP-SD composites displayed a good agreement between the FEA and experimental results and were validated against each other as shown in Figure 5. Similar observations were made on the stress- and strain-related analysis using ANSYS software on different WPCs. Values were evaluated and validated experimentally and using ANSYS [70,71].

#### 3.3.2. Deformation

**Figure 6:**
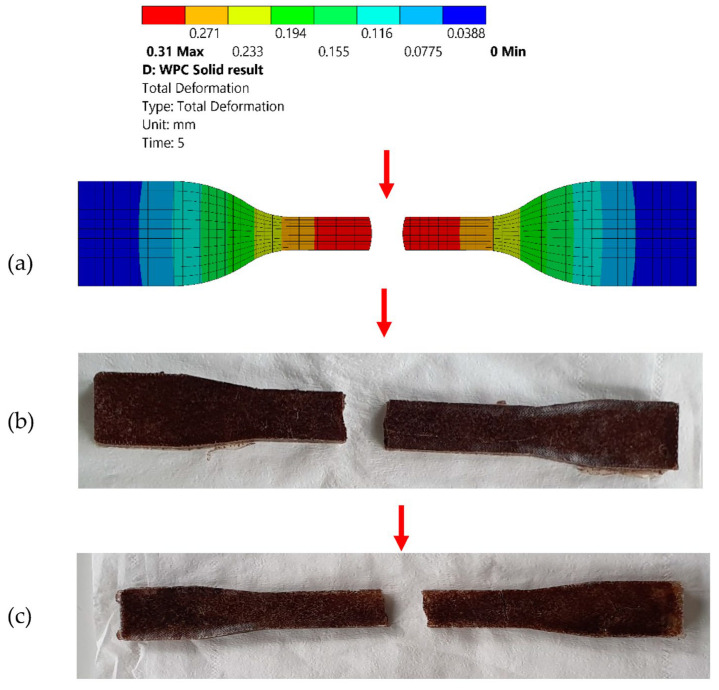
Total deformation in the (**a**) FEA, (**b**) rPP-SD3 and (**c**) rPP-SD4.

The total deformation of the specimen occurred at a higher concentration area, shown with a red arrow in Figure 6a. The variation in deformation for the experimental and simulation values was 0.54 mm for rPP-SD3 and 0.68 mm for rPP-SD4. This variation in deformation can be considered as the tolerance of the machine [18]. Fang et al. [72] studied the mechanical performance of glass fiber-reinforced polymer (GFRP)–bamboo wood sandwich beams. The beams were investigated experimentally and validated using ANSYS Workbench. The experimental results showed that with the increase in the thickness of the bamboo and GRPF layers, there was a significant increase in the tensile strength and deformation capacity of the beams. Furthermore, the experimental deformation results were considerably similar to the FEA. The deformation and structural analysis of a sisal fiber-reinforced polymer composite-based wind turbine were investigated by Appadurai et al. [73]. In their study, the wind turbine profile was modelled in CATIA V5 (Dassault Systèmes SE, Vélizy-Villacoublay, France) and evaluated for its mechanical properties in ANSYS. The results stated that the sisal fiber-reinforced polymer composite-based wind turbine had superior mechanical properties compared to structural steel and other natural fiber composite wind turbines. Rostampour-Haftkhani et al. [74] measured and predicted the deformation performance of WPC profiles. The deformation of the WPC profiles was analyzed experimentally and with ANSYS software in order to decrease the cost and time of measurement. The results stated that FEA predicted deformation values with a mean absolute percentage of less than 3% and it was suggested as an efficient method for predicting the deformation properties of WPC profiles. In conclusion, the simulation result correlates with the experimental result as shown in Figure 6b,c, as it has a similar fracture concentration area and exhibits the same deformation rate.

## 4. Conclusions

This study aimed to investigate the mechanical properties of sawdust-reinforced recycled polypropylene composites. The morphology of the composites showed a homogeneous dispersion of SD in rPP, which aided in ensuring good mechanical properties. Von Mises stress, equivalent elastic strain and deformation were investigated. The results showed that the stress concentration region was due to the force applied according to simulation parameters. Similarly, the strain rate depended on the thickness and the force applied, while the deformation rate depended on the elongation of the specimen with respect to the stacked layers and the applied force. The location of the fracture point was predicted using FEA analysis. The analysis was validated using experimental data. Both the FEA and experimental results showed a similar trend in the stress and strain plot. Furthermore, the orientation of the fibers and damage locations need to be further studied to obtain a better understanding and develop an accurate analytical model.

## Figures and Tables

**Table 1:** Composition of rPP-SD composites.

Composites	rPP (wt.%)	SD (wt.%)
rPP-SD3	70	30
rPP-SD4	60	40

**Table 2:** Properties of recycled polypropylene and sawdust.

Properties	SD	rPP
Young’s modulus (MPa)	0.30 [48]	6 [49]
Poisson’s ratio	0.33 [48]	0.38 [49]
Ultimate tensile strength (MPa)	0.5 [50]	21 [49]

## Data Availability

Not applicable.
